# Predictive Capacity of Beat-to-Beat Blood Pressure Variability for Cardioautonomic and Vascular Dysfunction in Early Metabolic Challenge

**DOI:** 10.3389/fphar.2022.902582

**Published:** 2022-06-24

**Authors:** Souha A. Fares, Nour-Mounira Z. Bakkar, Ahmed F. El-Yazbi

**Affiliations:** ^1^ Rafic Hariri School of Nursing, American University of Beirut, Beirut, Lebanon; ^2^ Department of Biostatistics and Informatics, Colorado University Anschutz Medical Campus, Aurora, Colorado; ^3^ Department of Pharmacology and Toxicology, Faculty of Medicine, American University of Beirut, Beirut, Lebanon; ^4^ Department of Pharmacology and Toxicology, Faculty of Pharmacy, Alexandria University, Alexandria, Egypt; ^5^ Faculty of Pharmacy, Alamein International University, Alalamein, Egypt

**Keywords:** blood pressure variability, approximate entropy, self-correlation, metabolic challenge, autonomic dysfunction

## Abstract

Diabetic patients present established cardiovascular disease at the onset of diagnostic metabolic symptoms. While premature autonomic and vascular deterioration considered risk factors for major cardiovascular complications of diabetes, present in initial stages of metabolic impairment, their early detection remains a significant challenge impeding timely intervention. In the present study, we examine the utility of beat-to-beat blood pressure variability (BPV) parameters in capturing subtle changes in cardiac autonomic and vascular control distinguishing between various risk categories, independent of the average BP. A rat model of mild hypercaloric (HC) intake was used to represent the insidious cardiovascular changes associated with early metabolic impairment. Invasive hemodynamics were used to collect beat-to-beat BP time series in rats of either sex with different durations of exposure to the HC diet. Linear (standard deviation and coefficient of variation) and nonlinear (approximate entropy, ApEn, and self-correlation of detrended fluctuation analysis, α) BPV parameters were calculated to assess the impact of early metabolic impairment across sexes and feeding durations. HC-fed male, but not female, rats developed increased fat:lean ratio as well as hyperinsulinemia. Unlike linear parameters, multivariate analysis showed that HC-fed rats possessed lower ApEn and higher α, consistent with early changes in heart rate variability and blunting of parasympathetic baroreceptor sensitivity, particularly in males. Moreover, logistic regression demonstrated the superiority of nonlinear parameters of diastolic BPV in predicting a prediabetic disease state. Our findings support the use of nonlinear beat-to-beat BPV for early detection of cardiovascular derangements in the initial stages of metabolic impairment.

## Introduction

Average blood pressure (BP) values have long been used to characterize overt changes in vascular function and BP control mechanisms associated with hypertension. However, a recent understanding of cardiovascular signals emphasizes variability in cardiovascular parameters as an indicator of the cardiovascular and autonomic control of hemodynamics (Parati et al., 2013a). BP spontaneously fluctuates in the long, short, and very short term conferring week-to-week, diurnal and beat-to-beat BP variability (BPV), respectively (Parati et al., 2013b). Significantly, abnormal long- and short-term BPV are reported to detect hidden and early changes in various pathophysiological states, establish risk stratification within the same condition, and predict prognosis independent of mean systolic (SBP) and diastolic BP (DBP) (Hsu et al., 2016; Chowdhury et al., 2018; Palatini et al., 2019).

Continuous BPV captures the intricacies of BP dynamics which are not otherwise collected by intermittent BP monitoring (Webb and Rothwell, 2014; Wei et al., 2014; Webb et al., 2018). Linear and nonlinear parameters describe different aspects of beat-to-beat BPV. Linear time-domain parameters are mainly measures of dispersion frequently reported to increase in pathological conditions (Parati and Ochoa, 2019). Indeed, aberrant beat-to-beat BPV is associated with end-organ damage related to excursions in perfusion (Wei et al., 2014). Alternatively, nonlinear parameters quantify complexity and regularity that decreases and increases in disease states, respectively (Bakkar et al., 2021). Despite the value of linear parameters of variability, fluctuations of the cardiovascular system are described as nonhomogeneous, that is, different parts of the signal exhibit distinctive scaling properties, and are thus believed to be better quantified using nonlinear parameters (Ivanov et al., 2001). Indeed, entropy and detrended fluctuation analysis of the beat-to-beat BP time series are shown to possess superior power of discriminating among patient groups compared to conventional parameters of variability and average BP (Bakkar et al., 2021).

Research on beat-to-beat BPV remains a relatively new field with a potential early diagnostic value for premature changes prior to overt symptomatic manifestations (Wu et al., 2017). Particularly, studies of beat-to-beat BPV in the context of metabolic diseases remain limited (Bakkar et al., 2021), with previous studies, not directly measuring beat-to-beat BPV, in adults with metabolic syndrome, indicating possible changes in these parameters (Chang et al., 2016). Hence, it is prudent to study continuous BPV in transition states like prediabetes (Chang et al., 2016) and prehypertension (Pal et al., 2015) and its sex-specific evolution over time.

In this regard, we demonstrated in our previous work that 12 weeks of mild hypercaloric (HC) feeding led to subtle metabolic impairment characterized by hyperinsulinemia, hyperlipidemia, and altered body composition in the absence of hyperglycemia, hypertension, and increased body weight recapitulating the early stages of prediabetes (Al-Assi et al., 2018; Alaaeddine et al., 2019; Elkhatib et al., 2019; Fakih et al., 2020; Hammoud et al., 2021a). Such metabolic alterations were associated with cardiac-, renal-, and cerebral-vascular dysfunction as well as cardiac autonomic deterioration only discernible upon considerably invasive hemodynamic interventions. Nevertheless, our results demonstrated the ability of nonlinear metrics of beat-to-beat BPV to describe the progression from prediabetes to type 2 diabetes with worsening of baroreflex function (Bakkar et al., 2020). We also showed the ability of nonlinear metrics to discriminate between hypertensive and nonhypertensive rats switching from low- to high-salt diet while linear parameters remained unchanged across groups and experimental conditions (Fares et al., 2016).

Our present study aimed to examine the role of BPV as a novel cardiovascular risk factor discriminating between subjects as to the presence or absence of a subtle underlying vascular pathology. For this purpose, we utilized our established model of mild metabolic challenge as a representation of the transition state of early cardiometabolic dysfunction to offer the much-needed pathophysiological insight. An implied hypothesis is that rats exposed to the mild HC challenge will exhibit different BPV profiles evolving disparately with sex over time, irrespective of mean BP. We also aimed to compare the powers of linear and nonlinear BPV parameters in predicting this early altered metabolic state.

## Methods

### Ethical Approval

Experimental procedures were performed according to a protocol approved by the Institutional Animal Care and Use Committee at the American University of Beirut in accordance with the Guide for Care and Use of Laboratory Animals of the Institute for Laboratory Animal Research of the National Academy of Sciences (National Research Council (US) Committee for the Update of the Guide for the Care and Use of Laboratory Animals, 2011).

### Experimental Design

Beat-to-beat BP time series were derived from two groups of Sprague–Dawley rats: a control group (*N* = 28) fed with a normal chow containing 3 kcal/g and HC-fed group (*N* = 23) receiving a HC diet providing 4.035 kcal/g. The normal chow contained 32% of calories from protein, 14% from fat (0.9% of weight saturated fat), and 54% from carbohydrates, while the HC diet was prepared in-house, as described previously, to contain 15.66% of calories from protein (15.8% by weight), 38.68% from fat (18.06% by weight of which 5% was saturated fat), and 45.73% from carbohydrates (46.13% by weight) (Al-Assi et al., 2018; Alaaeddine et al., 2019; Elkhatib et al., 2019; Fakih et al., 2020; Hammoud et al., 2021a). Rats of both sexes (24 males and 27 females) were received at 5 weeks of age, housed individually at standard temperature and humidity conditions with a 12-h dark/light cycle, and randomly divided into control and HC groups fed the corresponding diet *ad libitum* for a duration of either 12 weeks (22 rats) or 24 weeks (29 rats). The selection of feeding duration was based on our previous results where the end of week 12 was the earliest time point at which manifestations of metabolic dysfunction, which mimic prediabetes, started to appear in our rat model (Elkhatib et al., 2019). Particularly, hyperinsulinemia and dyslipidemia as well as perivascular adipose tissue inflammation started at week 12 (Al-Assi et al., 2018; Elkhatib et al., 2019). Signs of early cardiovascular (Alaaeddine et al., 2019; Elkhatib et al., 2019), cerebrovascular (Fakih et al., 2020), renovascular (Hammoud et al., 2020; Al-Saidi et al., 2021) deteriorations, as well as cardiac autonomic neuropathy (Al-Assi et al., 2018), were also evident at 12 weeks. No signs of overt cardiac or vascular dysfunction manifested up to 12 weeks of HC feeding, as indicated by echocardiography and noninvasive blood pressure monitoring performed biweekly starting from baseline (Al-Assi et al., 2018). Starting week 24, our data showed that fasting and random blood glucose as well as HbA1C levels begin to rise gradually, possibly marking the progression to type 2 diabetes (Elkhatib et al., 2019; Fakih et al., 2020). As such, we have chosen these two time points to represent compensated versus decompensated metabolic disease states in an attempt to assess the diagnostic, and rather discriminatory, capacity of beat-to-beat BPV parameters in this context (Bakkar et al., 2020).

Our experimental design targeted a typical group size of 5–7 animals shown to yield enough statistical power and an allocation strategy described in detail in our previous work (Elkhatib et al., 2019). The number of control female rats fed for 24 weeks was exceptionally duplicated due to an ordering error that occurred during the period of pandemic-related lockdowns. To avoid potential selection bias, their results were added. At the end of the designated feeding duration, the control group comprised 12 male rats (six 12-week and six 24-week) and 16 female rats (five 12-week and eleven 24-week), while the HC group had 12 male rats (six 12-week and six 24-week) and 11 female rats (five 12-week and six 24-week). Calorie intake was determined for each rat based on the amount of food consumed daily. Anesthetized rats were sacrificed by decapitation after 12 (age = 17 weeks) or 24 weeks (age = 29 weeks) of feeding (Dwaib et al., 2020).

### Echocardiography

In order to assess the heart structure and function, echocardiography along the parasternal long axis M- and B-modes was performed 1 day before sacrifice using SonixTouch Q+ ultrasound (BK ultrasound, Peabody, MA, United States) on rats sedated with a mixture of ketamine and xylazine (80% of 1.5 mg/ml/kg of ketamine followed by 80% 0.375 mg/ml/kg of xylazine for complete sedation). Images were acquired at a probe frequency of 20.0 MHz. For structural left ventricular (LV) parameters, interventricular septum thickness (during systole and diastole), posterior wall thickness (during systole and diastole), and LV diameters (during systole and diastole) were measured. LV end systolic volume, end diastolic volume, as well as LV mass were automatically estimated based on experts’ recommendations (Belenkie et al., 1973; Gibson, 1973; Teichholz et al., 1976; Oh et al., 2006). All structural indices were normalized to body weight and tibia length. As for functional parameters, the device automatically calculates ejection fraction (EF), fractional shortening, and stroke volume, according to standard formulas from Terry Reynold’s *The Echocardiographer’s Pocket Reference*.

### Body Composition Analysis, Blood Glucose Levels, and Serum Insulin Concentrations

The rat fat:lean ratio was measured using the LF10 Minispec nuclear magnetic resonance (NMR) machine (Bruker, MA, United States) detecting different tissue densities as previously described (Fakih et al., 2020). On the other hand, similar to our previous work (Bakkar et al., 2020), random blood glucose levels (BGLs) were measured using an Accu-Chek glucometer (Roche Diagnostics, Basel, Switzerland) by lateral tail vein puncture on the day of sacrifice, before anesthesia induction for the surgical procedure. Serum insulin concentrations were measured using ELISA kit (Cat. no. ERINS), according to the manufacturer’s protocol (Thermo Scientific, Walter, MA, United States).

### Invasive Hemodynamic Recording in Anesthetized Rats

Rats were anesthetized and instrumented for invasive hemodynamic monitoring as previously described (Al-Assi et al., 2018; Bakkar et al., 2020). Briefly, rats were intraperitoneally injected with thiopental (50 mg/kg) to induce light anesthesia, followed by phenobarbital (10 mg/kg) for maintenance. A paw pinch test was performed prior to surgical intervention to confirm total loss of sensation. A similar dose of barbiturate was shown to preserve mechanisms of cardiovascular modulation, like BPV, HRV, vasopressor and vasodepressor responses, as well as baroreceptor sensitivity (BRS) (Yang et al., 1996; Bencze et al., 2013). It is thus believed to be suitable for use in hemodynamic experiments (Kuo et al., 2005; Bencze et al., 2013). Importantly, such a level of anesthesia was shown to maintain BP levels within the same range previously recorded invasively in conscious rats of the same model using the same experimental setup (Elkhatib et al., 2019). Prior to carotid catheterization, tracheostomy was performed in order to facilitate ventilation and prevent overaccumulation of respiratory secretions (Yang et al., 1996; Kuo et al., 2005). BP signals were obtained using a pressure transducer (SP844, Cat. no. 32030, MEMSCAP, Norway) connected to the carotid catheter. Acquiring beat-to-beat BP signals under such conditions (carotid catheterization of anesthetized rats) is an alternative to a more complex and elaborate procedure (involving tunneling and femoral catheterization) requiring extensive manipulation. While the effect of anesthesia on beat-to-beat BPV cannot be underestimated, the latter puts the rat under increased stress and infection risk, two factors which might affect beat-to-beat BPV and introduce confounders to its analysis.

PowerLab (Model ML870, AD Instruments Ltd., Dunedin, New Zealand) was used for data acquisition and LabChart Pro 8 (AD Instruments Ltd., Dunedin, New Zealand) software for recording. After stabilization and prior to BRS assessment, 25 min of signal acquisition was conducted. Then, SBP and DBP recordings for a stable 5-min time series were acquired at a sampling rate of 1,000 Hz corresponding to approximately 300,000 data points. At such a sampling rate, every heart beat would be represented by 150–200 data points. BP time series were then extracted and downsampled by 20× (50 Hz) to obtain approximately 15,100 data points (8–10 data points/heart beat) for beat-to-beat BPV analysis.

### Baroreceptor Sensitivity

BRS was determined using the vasoactive method as described previously (Al-Assi et al., 2018; Bakkar et al., 2020). After 30 min of signal stabilization, rats were intravenously (through a catheterized jugular vein) injected with increasing doses of the vasopressor, phenylephrine (ICN Biochemicals) (PE 0.25, 0.5, 0.75, 1, and 2 μg), followed by increasing doses of the vasodepressor, sodium nitroprusside (Sigma-Aldrich, 228710-5G) (SNP 0.5, 1, 2, 4, and 8 μg), in order to assess the functionality of the parasympathetic and the sympathetic arms of BRS in decreasing and increasing HR, respectively. Changes in the heart rate (ΔHR) were plotted as a function of the corresponding changes in mean arterial pressure (ΔMAP) in response to the vasoactive drugs. GraphPad Prism for Mac OS version 8 was used to calculate the BRS of the parasympathetic and sympathetic nervous system (PSNS and SNS, respectively) as the best-fit slope of the linear regression of ΔHR vs. ΔMAP in response to PE and SNP, respectively.

### Linear Beat-to-Beat HRV and BPV Measures

The standard deviation (SD) and coefficient of variation (CV) of beat-to-beat HR and BP values were calculated. SD was determined as a measure of dispersion around the mean, while CV was used to reduce the dependence of SD on the mean value (Di Rienzo et al., 1983; Jinadasa et al., 2018).

### Approximate Entropy Analysis

Approximate entropy (ApEn) was computed using a MATLAB (Mathworks, Natick, MA, United States) code derived in our laboratory. ApEn was determined as a measure reflecting system complexity or self-similarity in a time series (Bakkar et al., 2020). By definition, ApEn is the negative natural logarithm of the conditional probability that a sequence similar for *m* values remains similar at the next point within a tolerance *r* (Pincus, 1991; Pincus and Goldberger, 1994). ApEn was calculated for *m* = 2 and *r* = 0.2, where *r* is conventionally recommended to be in the range of (0.1–0.25) times the standard deviation of the given time series (Pincus, 1991; Chon et al., 2009). In our control rats, the SD of the systolic and diastolic BP time series ranged from ∼1.5 to 4, thus implicating a potential *r* value ranging from 0.15 to 1. Based on our previous work (Fares et al., 2016), we expected a decrease in entropy measures in disease states, and thus we opted for an *r* value toward the lower end of the range (0.2) to maximize ApEn value and increase the sensitivity of detection of differences among different groups. Importantly, at a heart rate of approximately 300 BPM, a series length of *L* = 1,500 cardiac beats and *N* = 15,100 data points were used for ApEn calculations. It is worth mentioning that the use of such a lengthy time series overcomes the potential “bias toward regularity” associated with ApEn (Bakkar et al., 2021). Indeed, as mentioned by Porta et al. (2007), the bias of counting self-matches is particularly a concern in short signals consisting of a “few hundreds of samples” or around 300 beat-to-beat samples (Porta et al., 2007), which is far below the number of samples we are considering. As such, the contribution of self-matches becomes minimal.

### Detrended Fluctuation Analysis

Detrended fluctuation analysis (DFA) is a measure of system correlation (Peng et al., 1995) on varying ranges (short- and long-term) (Fares et al., 2016). Briefly, the original time series is integrated and detrended and the root mean square fluctuation of the detrended time series is calculated over different box sizes (*n*). A correlation coefficient, α, is then calculated as the linear slope of the logarithm of root mean square fluctuation versus the logarithm of box size (*n*). α quantifies fractal scaling of beat-to-beat signals and reflects long-term self-correlation in multicomponent systems (Goldberger et al., 2002). In fact, fractal scaling emerges from complex and rather nonlinear coupling processes (Goldberger et al., 2002). As such, the fractal scaling exponent, α, assesses temporal characteristics, that is, correlations, of a time series with nonlinear control mechanisms (Ivanov et al., 2001). Thus, while α in itself might not be a nonlinear value, it rather depicts changes in nonlinear processes, the assessment of which in the context of early metabolic disease was the intent of the present study. DFA correlations were computed, for the same series length of 1,500 cardiac beats (5×300BPM) sampled at 50 Hz (15,100 data points), using a MATLAB code developed in our laboratory (Fares et al., 2016).

### Statistical Analysis

Normality was tested using the Shapiro–Wilk test. Continuous variables and variability metrics were summarized using means and standard errors (SEs) and were compared univariately across groups using the independent samples *t*-test or the Mann–Whitney *U* test. Three-way ANOVA was used for subgroup analysis to compare the changes in different parameters across dietary groups for different sexes and feeding durations. Sidak’s multiple comparison test was used for *post hoc* comparisons. Bootstrap multivariable linear regression models were conducted to determine the adjusted associations among sex, diet type and duration, and average BP and BPV metrics to test their interactions with independent variables irrespective of the average BP. Due to the small sample size, the nonparametric bootstrap technique was used to estimate SEs, 95% confidence intervals (CIs), and *p*-values for the regression coefficients. In the bootstrap analysis, 1,000 samples of the same size as the original sample were drawn, with replacement from the original data set. Logistic regression was carried out to determine and compare the powers of BPV parameters in predicting disease state, represented by the presence or absence of HC feeding. Odds ratio (OR), SEs, and 95% CIs were reported. All tests were two-tailed, and *p*-values < 0.05 were considered significant. Statistical analyses were performed using Stata version 13.1 for Windows and GraphPad Prism for iOS.

## Results

### Metabolic Impact of HC Feeding

**FIGURE 1:**
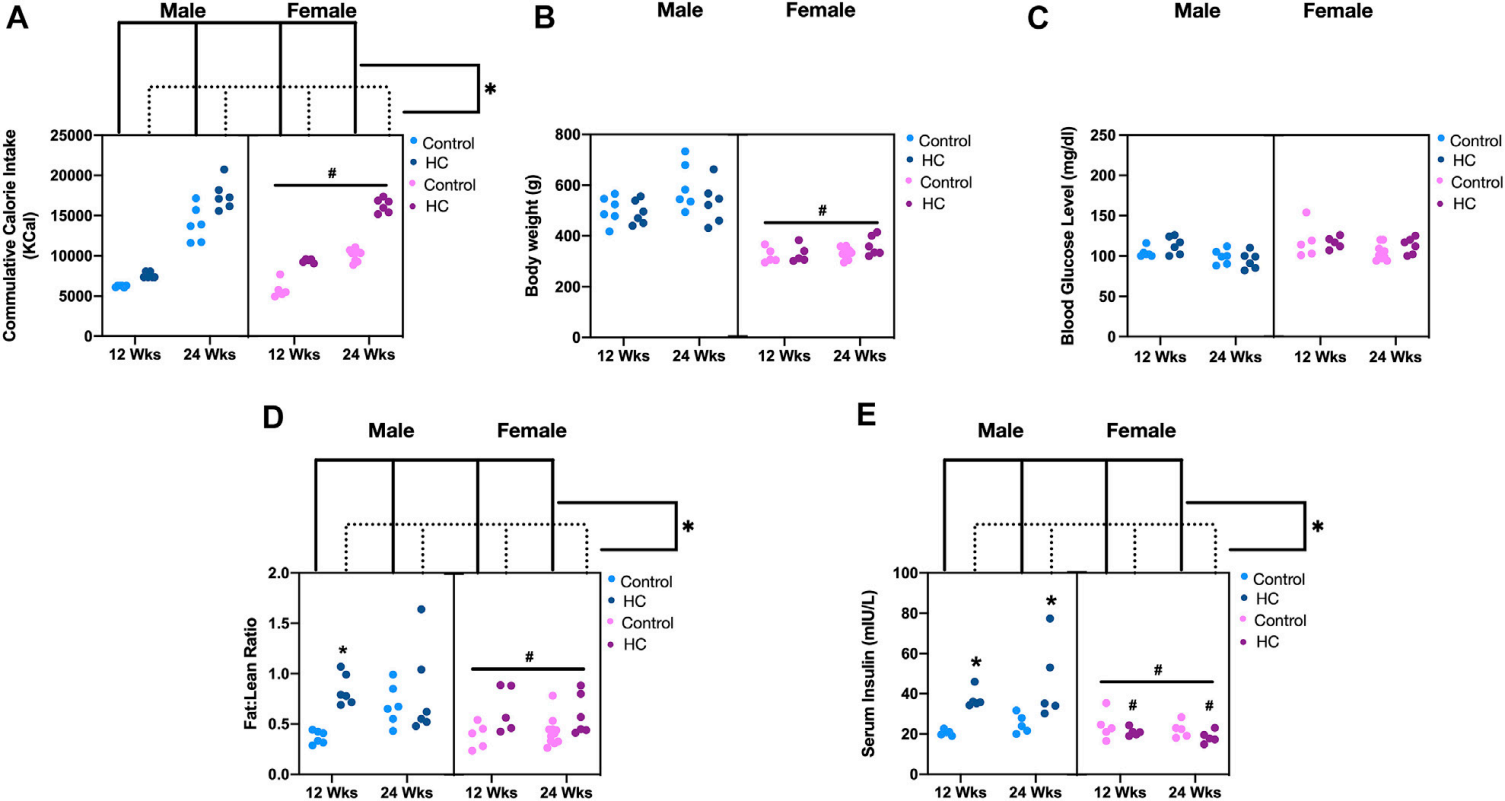
Metabolic parameters of different rat groups. Cumulative calorie intake **(A)**, body weight **(B)**, blood glucose level **(C)**, fat:lean ratio **(D)**, and serum insulin concentration **(E)** in control and hypercaloric diet (HC)-fed rats of either sex in different feeding duration groups. Statistical analysis was performed by three-way ANOVA followed by Sidak’s multiple comparison test. * denotes *p* < 0.05 vs. control group of the same sex and feeding duration group (control vs. HC), while # denotes *p* < 0.05 vs. the corresponding male group (males vs. females).

As expected, HC feeding for different durations was associated with increased caloric intake compared to the corresponding rat group receiving control diet, albeit being generally less in female compared to male rats (Figure 1A). Similar to our previous results (Al-Assi et al., 2018; Elkhatib et al., 2019; Fakih et al., 2020; Hammoud et al., 2021a), the increased calorie intake in HC-fed rats was not associated with an increase in neither body weight (Figure 1B) nor random blood glucose level (Figure 1C), yet HC-fed rats generally showed altered body composition as depicted by an increased fat:lean ratio across sexes and along different feeding durations (Figure 1D) confirming the occurrence of the previously demonstrated metabolic impairment. Interestingly, commensurate with the lower caloric intake in female rats, both their body weight and fat:lean ratio were lower than those of the male rat groups (Figures 1B,D). On the other hand, HC feeding was globally associated with hyperinsulinemia, mainly driven by significant increases in serum insulin levels in male rats fed HC diet for 12 or 24 weeks compared to their NC-fed counterparts (Figure 1E). Importantly, HC-fed female rats exhibited significantly lower serum insulin levels than their male counterparts.

### Gross Myocardial and Cardiac Autonomic Function Changes

**FIGURE 2:**
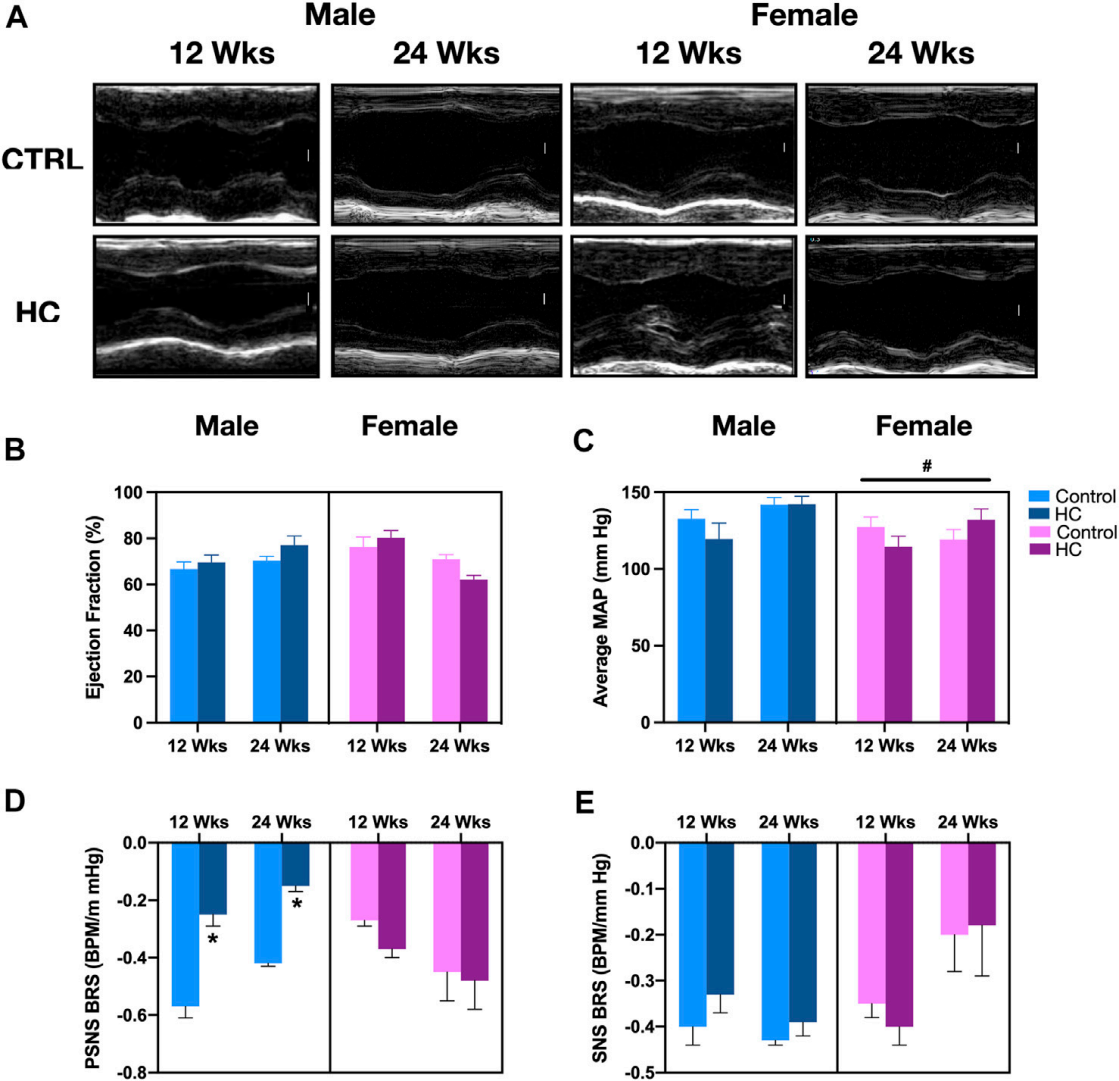
Gross myocardial, hemodynamic, and cardiac autonomic functions in response to HC-feeding as a function of feeding duration and sex. **(A)** Representative electrocardiograms of M-mode images (scale bar corresponds to 1 mm), **(B)** ejection fraction (EF) assessed by echocardiography, **(C)** average mean arterial pressure (MAP) assessed by invasive carotid artery catheterization, and slope of the best-fit linear regression of reflex bradycardic responses to a rise in mean arterial pressure (MAP) following increasing doses of phenylephrine (PE) or sodium nitroprusside (SNP) reflecting **(D)** parasympathetic nervous system (PSNS) or **(E)** sympathetic nervous system (SNS) activity, respectively, of control and hypercaloric diet (HC)-fed rats of either sex in different feeding duration groups. Statistical analysis was performed by three-way ANOVA followed by Sidak’s multiple comparison test. * denotes a *p* < 0.05 vs. control of the same sex and feeding duration group (control vs. HC), while # denotes a *p* < 0.05 vs. the corresponding male group (males vs. females).

Similar to our previous studies (Al-Assi et al., 2018; Bakkar et al., 2020), no changes were detected in baseline hemodynamics and myocardial structure or function across different diet groups. Indeed, Figure 2A shows the representative echocardiographic images without alteration in ejection fraction (Figure 2B). Similarly, there was no difference in MAP among control and HC rats of either sex or feeding duration group, albeit with female rats showing a lower MAP (Figure 2C). Significantly, an impaired cardiac autonomic activity, particularly on the parasympathetic side, was revealed following a hemodynamic challenge using a vasopressor agent. This was observed as a reduced parasympathetic BRS following HC feeding in both 12-week and 24-week male rats, but not in female rats (Figure 2D), and not in the sympathetic arm of baroreflex (Figure 2E). Consistently, a significant interaction appeared between sex and diet (*p* = 0.0003), whereby males were more prone to the effect of diet on parasympathetic BRS.

### Linear and Nonlinear Heart Rate Variability Indices

**FIGURE 3:**
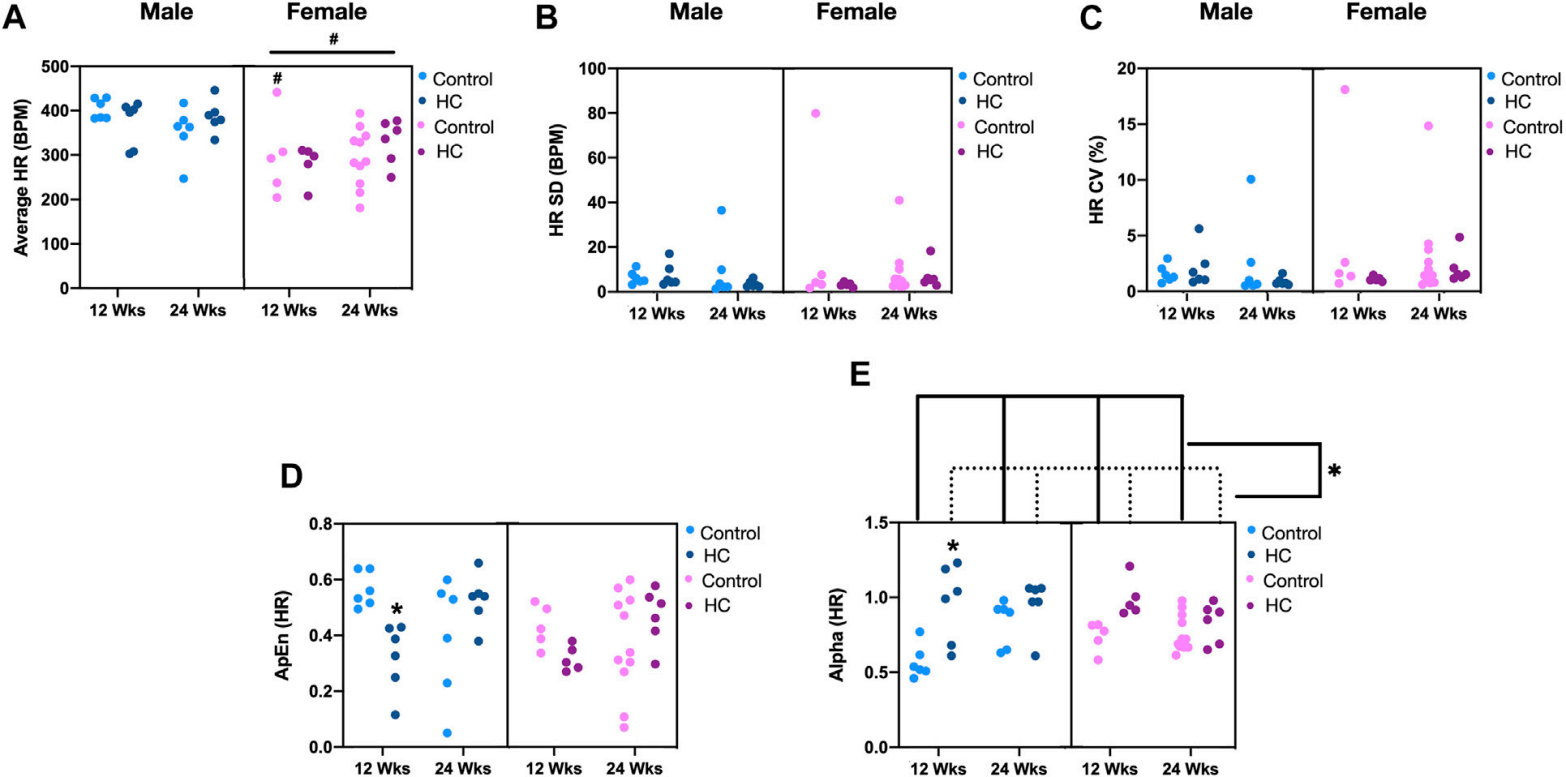
Impact of HC feeding on average HR and linear and nonlinear parameters of beat-to-beat HRV in rats of either sex in different feeding duration groups. **(A)** Average, **(B)** standard deviation (SD), **(C)** coefficient of variation (CV), **(D)** approximate entropy (ApEn), and **(E)** α of detrended fluctuation analysis of HR in control and HC-fed rats of either sex in different feeding duration groups. Statistical analysis was performed by three-way ANOVA followed by Sidak’s multiple comparison test. * denotes a *p* < 0.05 vs. control of the same feeding duration and sex (control vs. HC), while # denotes a *p* < 0.05 vs. the corresponding male group (males vs. females).

Examination of beat-to-beat HR tracing revealed that female rats collectively exhibited significantly lower average HR than males (Figure 3A). Particularly, healthy female rats demonstrated significantly lower average HR than their male counterparts (NC-fed for 12 weeks). Regarding HRV, no significant differences in linear fluctuations of HR, assessed by SD and CV, were observed among rats from either sex, on either diet for the different feeding durations (Figure 3B). Interestingly, alterations in nonlinear parameters of beat-to-beat HRV were exclusively observed in male rats on HC diet for 12 weeks, reflected by a decrease in ApEn and an increase in α of DFA with respect to their NC-fed counterparts (Figures 3C,D, respectively). Additionally, HC-fed rats collectively exhibited higher α of DFA than those fed an NC diet (Figure 3D). Importantly, significant interactions existed between feeding duration and diet for ApEn (*p =* 0.0004) and α (*p =* 0.014) of HR, indicating a more pronounced decrease and increase, respectively, in 12-week HC-fed rats. Another significant interaction was found between sex and feeding duration for α of HR (*p =* 0.027), reflecting a stronger effect of feeding duration on males.

### Linear Beat-to-Beat Blood Pressure Variability Indices

**FIGURE 4:**
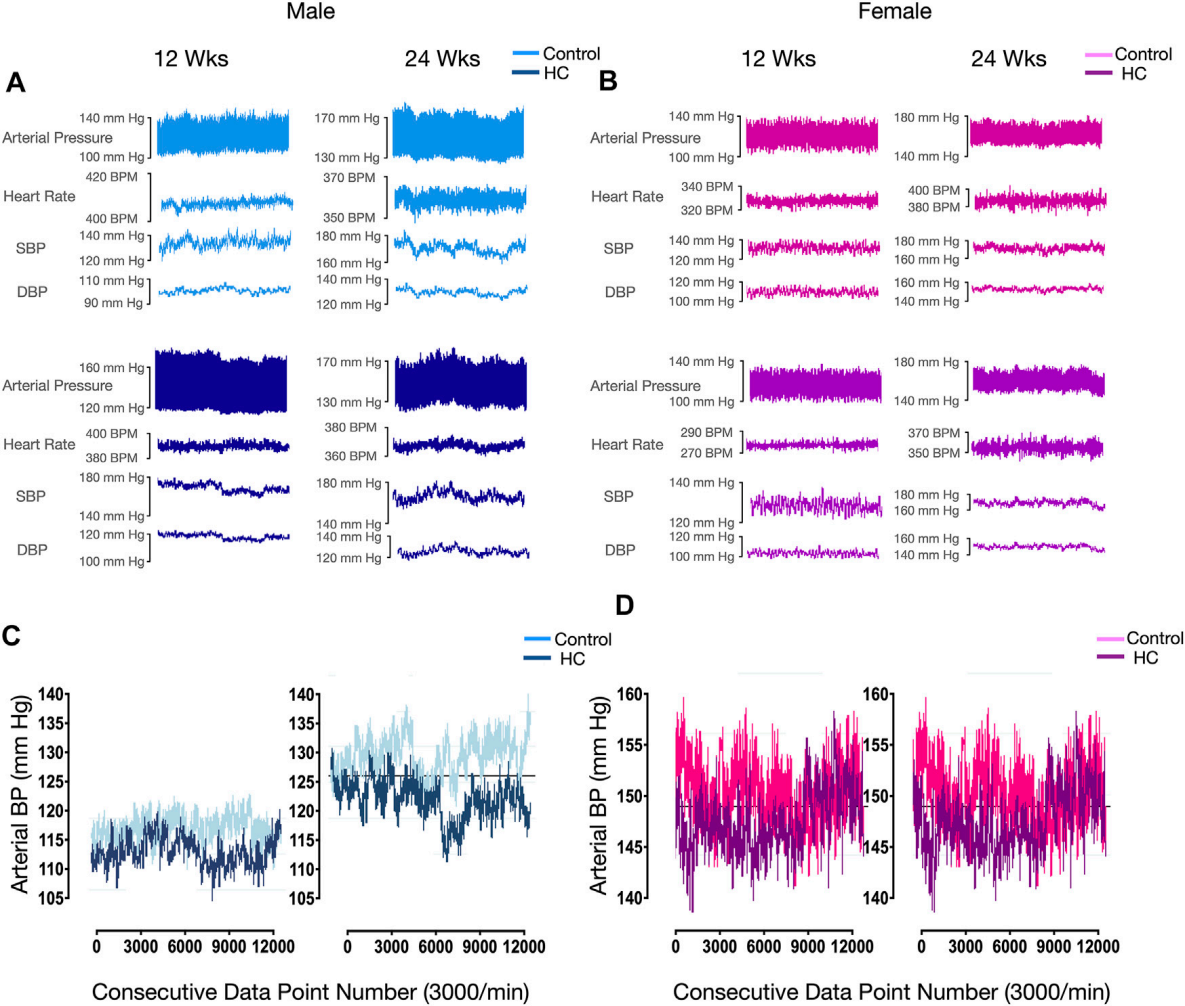
Representative beat-to-beat blood pressure and heart rate tracings of selected rats. Raw arterial pressure, heart rate, and systolic and diastolic blood pressure recordings in 12-week and 24-week control and HC-fed **(A)** male and **(B)** female rats. **(C)** Downsampled beat-to-beat diastolic blood pressure (BP) tracing of control and HC-fed male rats of different feeding duration groups showing equal average, SD, and CV values for all groups with reduced ApEn values in 12-week HC-fed rats. **(D)** Beat-to-beat systolic blood pressure (BP) tracing of control and HC-fed female rats of different feeding duration groups showing equal average, SD, and CV values for all groups with reduced ApEn and α values compared to male rats.

**FIGURE 5:**
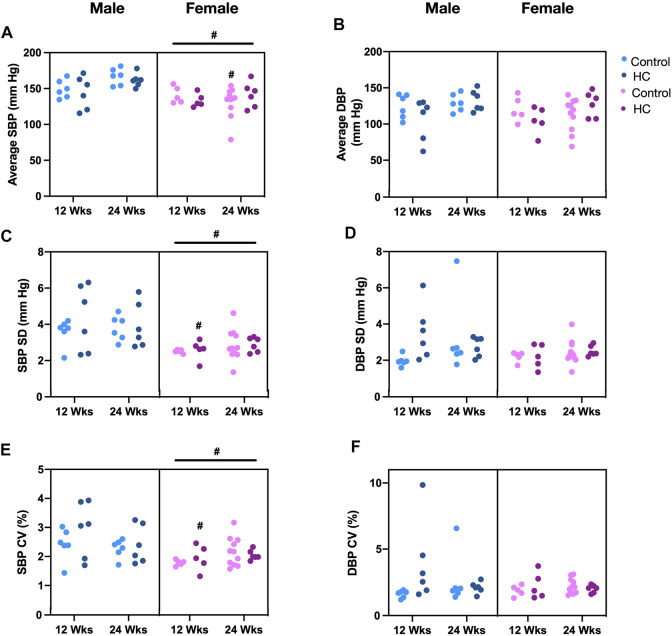
Impact of HC-feeding on average and linear parameters of beat-to-beat systolic and diastolic blood pressure (SBP and DBP, respectively) in rats of either sex in different feeding duration groups. **(A,B)** Average, **(C,D)** standard deviation (SD), and **(E,F)** coefficient of variation (CV) of SBP and DBP, respectively, in control and HC-fed rats of either sex in different feeding duration groups. Statistical analysis was performed by three-way ANOVA followed by Sidak’s multiple comparisons test. * denotes a *p* < 0.05 vs. control of the same feeding duration and sex (control vs. HC), while # denotes a *p* < 0.05 vs. the corresponding male group (males vs. females).

Figure 4 depicts BP tracings of the raw (Figures 4A,B) and downsampled signals (Figures 4C,D). Examination of beat-to-beat BP tracings revealed that despite equal average BP values, rats from different groups might demonstrate disparate variability patterns (Figure 4). Whereas female rats had lower average and linear BPV parameters for SBP, univariate analysis showed that the average beat-to-beat SBP, SD, and CV did not differ with respect to diet type and duration (Table 1). Other than an increased SD of DBP in HC-fed rats, no differences were observed in average DBP or CV for DBP by univariate analysis across sexes or with diet type or duration (Table 1). While further subgroup analysis using three-way ANOVA confirmed the broad changes across sexes for SBP parameters, 24-week control female rats had lower average SBP and 12-week HC-fed female rats showed lower values for SD and CV than their male counterparts (Figures 5A,C,E). No differences were observed for the average DBP or SD and CV values for DBP across sexes, diet type, or duration (Figures 5B,D,F).

**TABLE 1:** Univariate analysis of linear variability parameters of beat-to-beat systolic blood pressure(SBP) and diastolic blood pressure (DBP) time series across different rat groups. Statistical analysis was determined using *t*-test or Mann–Whitney *U* test based on the results of the Shapiro–Wilk test of normality. *denotes *p* < 0.05 for 12 weeks vs. 24 weeks, males vs. females, or control vs. HC.

Variable (*n*)		Mean		SD		CV	
		SBP	DBP	SBP	DBP	SBP	DBP
Feeding	12 weeks (22)	142.39 ± 3.29	114.34 ± 4.54	3.32 ± 0.26	2.50 ± 0.22	2.31 ± 0.16	2.43 ± 0.39
Duration	24 weeks (29)	146.9 ± 4.07	123.44 ± 3.57	3.28 ± 0.17	2.68 ± 0.20	2.21 ± 0.08	2.21 ± 0.18
Sex	Male (24)	155.44 ± 3.42	123.24 ± 4.16	3.91 ± 0.24	2.87 ± 0.28	2.51 ± 0.14	2.55 ± 0.40
	Female (27)	136.65 ± 3.23*	116.20 ± 3.91	2.75 ± 0.12*	2.37 ± 0.10	2.03 ± 0.075*	2.09 ± 0.11
Diet	Control (28)	144.45 ± 3.88	120.36 ± 3.56	3.16 ± 0.16	2.46 ± 0.21	2.18 ± 0.09	2.09 ± 0.19
	HC (23)	145.58 ± 3.81	118.48 ± 4.73	3.47 ± 0.27	2.77 ± 0.20*	2.35 ± 0.15	2.57 ± 0.37

SD, standard deviation; CV, coefficient of variation.

### Nonlinear Beat-to-Beat Blood Pressure Variability Indices

**FIGURE 6:**
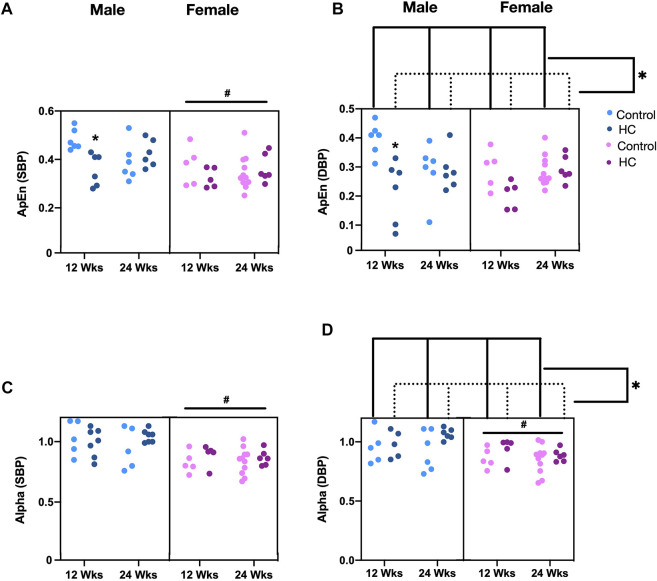
Impact of HC-feeding on nonlinear parameters of beat-to-beat systolic and diastolic blood pressure (SBP and DBP, respectively) in rats of either sex in different feeding duration groups. **(A,B)** Approximate entropy (ApEn) and **(C,D)** α of detrended fluctuation analysis of SBP and DBP, respectively, in control and HC-fed rats of either sex in different feeding duration groups. Statistical analysis was performed by three-way ANOVA followed by Sidak’s multiple comparisons test. * denotes a *p* < 0.05 vs. control of the same feeding duration and sex (control vs. HC), while # denotes a *p* < 0.05 vs. the corresponding male group (males vs. females).

Univariate examination of nonlinear BPV indices revealed a lower ApEn for SBP and α for both SBP and DBP in female rats compared to their male counterparts (Table 2). Moreover, a reduction and an increase in ApEn and α for DBP, respectively, were observed in HC-fed rats compared to those receiving the control chow (Table 2). Subgroup analysis confirmed a lower ApEn for SBP and α for both SBP and DBP in female rats (Figures 6A,C,D). For ApEn, while HC-feeding reduced the values for DBP across sex and feeding duration groups, 12-week HC-fed rats were particularly vulnerable to that effect for both SBP and DBP (Figures 6A,B). Indeed, there was a significant interaction between feeding duration and diet for ApEn of both SBP and DBP (*p =* 0.0041 and *p* = 0.01, respectively). On the other hand, α for DBP was increased for HC-fed rats across sex and feeding duration groups without a specific trend in one subgroup over the other (Figure 6D).

**TABLE 2:** Univariate analysis of nonlinear variability parameters of beat-to-beat systolic and diastolic blood pressure (SBP and DBP, respectively) time series across different rat groups. Statistical analysis was determined using *t*-test or Mann–Whitney *U* test based on the results of the Shapiro–Wilk test of normality. *denotes *p* < 0.05 for 12 vs. 24 weeks, males vs. females, or control vs. HC.

Variable (*n*)		ApEn		α	
		SBP	DBP	SBP	DBP
Feeding	12 weeks (22)	0.39 ± 0.017	0.28 ± 0.02	0.94 ± 0.03	0.96 ± 0.03
Duration	24 weeks (29)	0.37 ± 0.01	0.29 ± 0.01	0.91 ± 0.02	0.92 ± 0.02
Sex	Male (24)	0.41 ± 0.02	0.29 ± 0.02	1.00 ± 0.02	1.00 ± 0.29
	Female (27)	0.35 ± 0.01*	0.27 ± 0.1	0.85 ± 0.017*	0.88 ± 0.02*
Die**t**	Control (28)	0.39 ± 0.02	0.31 ± 0.01	0.90 ± 0.03	0.90 ± 0.03
	HC (23)	0.37 ± 0.01	0.25 ± 0.02*	0.96 ± 0.02	0.98 ± 0.02*

ApEn, approximate entropy; α, alpha of detrended fluctuation analysis.

### Adjusted Associations Between Study Variables and Linear Parameters of BPV Based on Bootstrap Linear Regression

Bootstrap multivariable linear regression models were conducted to determine the adjusted associations between average BP, sex, diet type and duration, and BPV metrics. Interactions among sex, and diet type and duration were examined to determine whether the associations with BPV metrics vary across the different rat categories, while that with average BP was performed to examine whether these parameters are affected by baseline BP. For the linear parameters, average SBP (B_SBP_ = 0.03, *p* < 0.001) and sex (B_SBP_ = 0.63, *p* = 0.014) were significantly associated with SD of the SBP and resulted in an adjusted *R* ^2^ of 0.3. As expected by the subgroup analysis, significantly higher values of SD were found in rats with higher average SBP and in males (Figures 5A,C), while no such associations were found with SD of the DBP. On the other hand, CV of the SBP series showed a significant interaction with feeding duration and sex resulting in an increase in CV in males only in the 12-week rats (B_SBP_ = 0.74, *p* = 0.008), possibly driven by the lower CV values observed in young HC-fed females (Figure 5E). The model, however, had an adjusted *R* ^2^ of 0.19 only. Average SBP was not associated with CV of the SBP. Alternatively, CV of the DBP was associated with average DBP (B_DBP_ = −0.42, *p* = 0.01) with an adjusted *R* ^2^ of 0.4. Surprisingly, a decrease in mean DBP was associated with an increase in CV.

### Adjusted Associations Between Study Variables and Nonlinear Parameters of BPV Based on Bootstrap Linear Regression

As opposed to findings related to SD for SBP, no association was observed for any of the nonlinear parameters with average BP. For ApEn regression models, significant interactions were found with diet type and duration for both SBP and DBP series (*p* = 0.005 and *p* = 0.005, respectively). Adjusted *R* ^2^ for the overall models with these interactions were 0.39 for SBP and 0.43 for DBP. After running separate models for 12-week and 24-week rats, ApEn was found to decrease in 12-week HC-fed rats only (B_SBP_ = −0.08, *p* = 0.005 and B_DBP_ = −0.1, *p* < 0.001) similar to observations in subgroup analyses (Figures 6A,B). No changes in ApEn across diets and sex were observed in the 24-week rats. No significant interactions were found with α of both BP series. Adjusted *R* ^2^ for the overall models were 0.36 for SBP and 0.23 for DBP. α was higher in males in both series (B_SBP_ = 0.13, *p* = 0.001 and B_DBP_ = 0.12, *p* = 0.001) (Figures 6C,D) and in DBP for HC-fed rats (B_DBP_ = 0.07, *p* = 0.039) (Figure 6D) similar to what was concluded from the subgroup analysis.

### BPV Parameters in Predicting Early Disease State

Since ApEn and α for DBP were found in subgroup analysis and bootstrap regression to consistently decrease and increase, respectively, in different rat groups following HC-feeding, logistic regression was carried out to determine and compare the powers of BPV parameters in predicting a prediabetic disease state induced by HC feeding and associated with subtle yet significant cardiovascular and autonomic impairment. The overall logistic regression model was significant (*p*-value = 0.0159, Pseudo *R* ^2^ = 17.4%). As expected, a 0.1 unit increase in ApEn or α of DBP was associated with an OR of 0.27 ± 0.16 (CI: 0.09–0.85, *p* = 0.026) or 1.69 ± 0.43 (CI: 1.03–2.79, *p* = 0.040), respectively, for the incidence of early cardiovascular involvement and metabolic impairment associated with HC feeding (Table 3). Linear parameters of DBP, SD and CV, did not significantly predict disease stage with ORs of 1.2 ± 0.77 (CI: 0.34–4.25, *p* = 0.773) and 0.7 ± 0.44 (CI: 0.21–2.4, *p* = 0.574) for a 0.1 unit increase in SD and CV of DBP, respectively.

**TABLE 3:** Odds ratio reflecting the potential predictive capacity of linear and nonlinear diastolic blood pressure variability parameters for early cardiovascular dysfunction associated with a mild metabolic challenge triggered by HC-feeding.

	**Based on a 1-unit increase in ApEn and α**		**Based on a 0.1-unit increase in ApEn and α**	
**BPV parameter**	**OR**	**SE**	**OR**	**SE**	***p*-value**	**95% CI**
ApEn	2 × 10^–6^	1.2 × 10^–5^	0.27	0.16	0.026	(0.09–0.85)
α	192.44	492.19	1.69	0.43	0.040	(1.03–2.79)
SD	1.20	0.77	1.20	0.77	0.773	(0.34–4.25)
CV	0.70	0.44	0.70	0.44	0.574	(0.21–2.40)

OR, odds ratio; SE, standard error; CI, confidence interval; ApEn, approximate entropy, α, alpha of detrended fluctuation analysis; SD, standard deviation; CV, coefficient of variation.

## Discussion

Despite significant advances in care for diabetic patients in the past decade, evidence has long indicated that glycemic control was not sufficient to reduce cardiovascular complications (Giorgino et al., 2013; Hayward et al., 2015; Barer et al., 2018). Indeed, the deleterious process culminating in overt cardiovascular dysfunction as diabetic patients progress along the course of the disease appears to start much earlier than the initial diagnosis of hyperglycemia, extending the elevated cardiovascular risk to the prediabetic stage (Fowler, 2008; DeFronzo and Abdul-Ghani, 2011; Wasserman et al., 2018). In this regard, one of the greatest challenges would be the detection of changes in cardiovascular function triggered by early metabolic impairment leading to increased risk of morbidity and mortality, especially since many of these changes are subtle or asymptomatic, and an onset point of metabolic impairment lacks an unequivocal definition. Here, we propose the use of nonlinear BPV parameters as potential predictors of the earliest signs of cardiovascular involvement in the course of metabolic disease. To the best of our knowledge, ours is the first study to assess the impact of early metabolic challenge on linear as well as nonlinear parameters of beat-to-beat BPV in a nonobese prediabetic model. While our experimental method collected BP data series using an invasive technique, calculation of such parameters is equally feasible using noninvasive recording methods (Bakkar et al., 2021).

In this context, we measured linear and nonlinear BPV parameters in control and HC-fed rats of either sex exposed to different feeding durations. Indeed, 12 weeks of mild hypercaloric challenge were shown to induce an early state of nonobese metabolic dysfunction characterized by hyperinsulinemia associated with increased fat:lean ratio and localized adipose tissue inflammation in male rats, while lacking hyperglycemia and signs of systemic inflammation (Al-Assi et al., 2018; Alaaeddine et al., 2019; Elkhatib et al., 2019; Fakih et al., 2020; Hammoud et al., 2021a). While these rats lacked signs of gross cardiovascular dysfunction, with no hypertension or cardiac remodeling seen with echocardiography, renal and vascular endothelial, cerebrovascular, and parasympathetic cardiac autonomic dysfunction demonstrated as suppressed measures of time-domain and frequency-domain parameters of heart rate variability (HRV) together with reduced BRS, as well as markers of cardiovascular oxidative stress and inflammation, were seen upon molecular profiling (Al-Assi et al., 2018; Alaaeddine et al., 2019; Elkhatib et al., 2019; Fakih et al., 2020; Hammoud et al., 2021a). Thus, we believe that HC-fed rats would serve as a reasonable model for mild metabolic impairment with early cardiovascular involvement lacking baseline gross diagnostic signs.

Importantly, the association between metabolic syndrome and cardiac autonomic dysfunction is well documented and thought to underlie the increased risk of incidence of other cardiovascular disorders (Fowler, 2008). In this regard, the study of the considerable and characteristic degree of change associated with various physiological signals such as heart rate (HR) and BP due to the competitive interaction between different reflex control mechanisms might offer a means of detecting autonomic dysfunction. While cardiorespiratory and baroreceptor reflexes reflect as variability in both HR and BP, a main advantage of BPV over HRV analysis is its capacity to depict an integrative view of vascular and autonomic control. Specifically, vascular properties like reactivity (Xu et al., 2017), elasticity, and compliance (Webb and Rothwell, 2014; Xia et al., 2017), together with autonomic input through BRS, were shown to affect beat-to-beat BP dynamics (DeBoer et al., 1987; Grilletti et al., 2018). As such, it became prudent to investigate the capacity of BPV parameters to reflect and predict early cardiovascular dysfunction in a prediabetic disease state (Chang et al., 2016).

Univariate analysis of our present results showed that changes in nonlinear BPV parameters indicative of cardiovascular pathology occurred in HC-fed rats more so than those in linear parameters. Indeed, a reduction in DBP beat-to-beat signal complexity, manifested as a lower ApEn, and increased self-correlation, measured as a higher α for DBP, were observed in HC-fed rats of either sex at different feeding durations. Only an increased beat-to-beat DBP SD was observed for HC-fed rats. Interestingly, multivariate subgroup analyses revealed that these changes persisted only for the nonlinear parameters, ApEn and α, but not for SD. Moreover, adjusted bootstrap analysis confirmed that, unlike linear parameters, nonlinear BPV parameters appeared to be affected by intake of HC diet and hence early metabolic impairment. Our results are in line with observations from different human studies revealing that various pathological conditions are associated with a similar trend of beat-to-beat BP aberrations (Bakkar et al., 2021).

Despite the fact that HRV is believed to be more complex owing to cardiac parasympathetic innervation and antagonistic firing (Porta et al., 2012), the paradigm of pathological increase in signal predictability, reflected by migration of ApEn and α to lower and higher values, respectively, typically observed with HRV (Goldberger et al., 2002), seems to hold for BPV. In fact, although beat-to-beat BPV is essentially under sympathetic control, vagal activity was found to have an indirect effect on BPV by modulating HRV. For example, Castiglioni et al. (2011) showed that parasympathetic outflow alters BP scaling exponents by increasing white noise in HR time series. As such, beat-to-beat BPV analysis is complementary to HRV assessment (Porta et al., 2012). Indeed, our study reveals similar HC-induced changes in nonlinear HRV and BPV in 12-week-fed male rats.

Interestingly, neither ApEn nor α seemed to be affected by BP as opposed to SD for SBP. Given the lack of difference in BP among control and HC-fed rats observed in our present as well as previous studies (Al-Assi et al., 2018; Alaaeddine et al., 2019; Elkhatib et al., 2019; Fakih et al., 2020; Hammoud et al., 2021a), this latter finding suggests that nonlinear parameters might be a more reliable reflection of the cardiovascular status in early metabolic impairment. Indeed, the values of α of DFA remained approximately equal to 1 in HC-fed rats indicating the presence of pink noise which typically describes healthy time series that are essentially complex with a degree of long-term self-correlation (Peng et al., 1995; Goldberger et al., 2002). However, slight yet significant differences in α of BPV were observed between the different groups according to their propensity to early cardiovascular impairment.

Significantly, logistic regression indicated a solid ability of nonlinear diastolic BPV parameters to predict the cardiovascular status associated with the mild metabolic impairment triggered by the HC diet. A 0.1 unit decrease or increase in DBP ApEn or α corresponded to an increased risk of such a state by 73% or 69%, respectively. This was not the case with DBP SD or CV as linear parameters of BPV. Such an outcome would not be surprising since nonlinear parameters are based on the postulation that erratic cardiovascular processes stem from competitive autonomic signals, which feed into a system whose components do not sum up to the whole (Subramaniam et al., 2014). In this regard, it is plausible that the altered sympathovagal balance observed in HC-fed rats (Al-Assi et al., 2018), together with the impaired endothelial function (Alaaeddine et al., 2019; Fakih et al., 2020) and increased sensitivity to vasoconstrictors (Elkhatib et al., 2019), have interacted to confer these changes in ApEn and α. Indeed, these latter observations suggest that HC-fed rats are prone to furnish an increased peripheral vascular resistance commonly reflected by DBPV as a surrogate measure (Carrara et al., 2018).

From a different perspective, the results of the present study showed that females exhibited a more favorable beat-to-beat BPV profile compared to males reflecting the sex-based disparity where females are postulated to require a more profound metabolic deterioration prior to significant cardiovascular risk involvement (de Ritter et al., 2020). Consistently, female rats seemed resistant to diet-induced hyperinsulinemia and fat accumulation and thus had preserved BRS and HRV. Univariate analysis revealed that females possessed lower average and linear fluctuations (SD and CV) of SBP and a lesser degree of self-correlation in SBP and DBP time series. Particularly, on subgroup comparisons, 24-week control female rats were shown to possess lower average SBP than males of the same group. Such observations could be possibly attributed to vascular and/or autonomic sex-specific characteristics. On the vascular level, α-adrenergic vasoconstriction was shown to be masked by β-adrenergic vasodilation in females (Hart et al., 2012). Additionally, vagal activity was found to predominate in females as opposed to sympathetic firing in males (Dart et al., 2002; Du et al., 2006).

On the other hand, sex segregation revealed that HC-induced alterations in beat-to-beat BPV were more pronounced in males. Along the same lines, diet-induced sympathovagal imbalance in response to HC feeding (Al-Assi et al., 2018; Hammoud et al., 2021b) can possibly explain sex differences in propensity to aberrations in vascular dynamics (Laitinen et al., 1999). Indeed, augmented sympathetic activity is associated with increased total peripheral resistance in men but not in women (Hart et al., 2012). The latter is concordant with the observation that changes in diastolic BPV significantly predicted prediabetic disease state. Consistent with our results, such a discrepancy was related to differential fat distribution along with less variable glucose homeostasis and kidney function in women (Barajas Martínez et al., 2021).

Women are more likely to store fat subcutaneously and on their lower extremities, whereas men are more likely to store fat in the abdominal region (Power and Schulkin, 2008). Not only is visceral adiposity associated with worse metabolic outcomes (Karelis et al., 2004), but it was also correlated to increased pro-inflammatory cytokine production (Beasley et al., 2009) potentially suggesting a worsened inflammatory-driven cardiovascular outcome in males. Significantly, estrogen receptor α deletion in mice was shown to increase diet-induced adipose inflammation, to which control mice were relatively resistant (Ribas et al., 2010). Moreover, estrogen signaling was found to produce antiadipogenic and anti-inflammatory effects in perivascular adipose tissues which were associated with amelioration of endothelial function (Sun et al., 2020).

Interestingly, diet-induced changes in BPV parameters appeared to be driven mainly by 12-week male rats despite a similar decrease in parasympathetic BRS in 24-week male rats compared to their control counterparts. This suggests that while BRS reflects cardiovascular involvement in prediabetic metabolic dysfunction, it correlates weakly with beat-to-beat BP dynamics with increasing age. This could be attributed to age-dependent increases and decreases in vascular stiffness and reactivity, respectively, which are stronger determinants of beat-to-beat BPV.

Results from our study show that different linear and non-linear parameters of beat-to-beat BPV possess varying sensitivities to sex and early metabolic impairment. Indeed, nonlinear parameters of beat-to-beat BPV, namely ApEn and α, are superior in predicting the early signs of cardiovascular and autonomic dysfunction. These results support the use of beat-to-beat BPV assessment as a screening tool for insidious autonomic and vascular dysfunction in early stages of metabolic conditions including prediabetes. Interestingly, our previous work indicates worsening control of beat-to-beat BPV on establishment of type 2 diabetes in the same rat model (Bakkar et al., 2020).

Significantly, a limitation of the present study is that the results reflect the processing of data collected by invasive techniques from anesthetized rats. Future work using telemetry recording methods has the potential to reveal more details on the diurnal changes of these parameters in early metabolic dysfunction and thus further highlight these differences. Additionally, direct assessment of sympathetic nerve activity has the capacity to provide insight into the pathophysiological determinants of beat-to-beat BPV changes in early metabolic disease (Kuo et al., 2005). Yet, the resilience of the detected differences in face of anesthesia emphasizes the potential diagnostic value of the nonlinear BPV metrics. Indeed, the availability of noninvasive methods to monitor beat-to-beat BP in patients, such as finger plethysmography and tonometry (Kemmotsu et al., 1991), makes the utility of beat-to-beat BPV attractive, especially in the light of studies indicating the validity of noninvasive techniques in comparison to invasive methods (Gibson et al., 2019).

Moreover, the discrepant effect of anesthesia in rats with different fat:lean ratios secondary to HC diet feeding cannot be precluded. Distribution of anesthesia into fat tissues can alter the final effective concentration in HC-fed rats (Brandon and Baggot, 1981). However, in our hands, rats from the different groups reached the same sedative levels before the surgical procedure. It is worth mentioning that studies have indicated that invasive, perioperative beat-to-beat BPV assessment under similar conditions (anesthesia and arterial catheterization) provides valuable information for risk appraisal, making such an assessment reasonably acceptable (Subramaniam et al., 2014; Jinadasa et al., 2018; Putowski et al., 2022). Extensive future work will be required for the appropriate translation of such findings to clinical practice.

## Data Availability

The original contributions presented in the study are included in the article/Supplementary Material; further inquiries can be directed to the corresponding author.
